# BRCA1 mRNA Expression as a Predictive and Prognostic Marker in Advanced Esophageal Squamous Cell Carcinoma Treated with Cisplatin- or Docetaxel-Based Chemotherapy/Chemoradiotherapy

**DOI:** 10.1371/journal.pone.0052589

**Published:** 2013-01-09

**Authors:** Yong Gao, Jing Zhu, Xiaohui Zhang, Qingquan Wu, Shaoning Jiang, Yangqing Liu, Zhibin Hu, Baorui Liu, Xiaofei Chen

**Affiliations:** 1 Department of Medical Oncology, Huai'an First People's Hospital, Nanjing Medical University, Huai'an, China; 2 Department of Thoracic Surgery, Huai'an First People's Hospital, Nanjing Medical University, Huai'an, China; 3 Department of Medicine, University of Alabama at Birmingham, Birmingham, Alabama, United States of America; 4 Department of Epidemiology and Biostatistics, Nanjing Medical University, Nanjing, China; 5 The Comprehensive Cancer Center, Gulou School of Clinical Medicine, Nanjing Medical University, Nanjing, China; Kinghorn Cancer Centre, Garvan Institute of Medical Research, Australia

## Abstract

**Background:**

The molecular backgrounds that determine therapeutic effectiveness in esophageal cancer remain largely unknown. Breast cancer susceptibility gene 1 (BRCA1) expression has been found to switch the response to cisplatin- or paclitaxel-based chemotherapy. It remains unclear how variations in BRCA1 expression influence clinical outcomes in esophageal cancer.

**Patients and Methods:**

Quantitative real-time polymerase chain reaction (qPCR) was performed to examine BRCA1 mRNA expressions in paraffin-embedded specimens from 144 patients with advanced or metastatic esophageal squamous cell carcinoma who received cisplatin- or docetaxel-based first-line treatments.

**Results:**

Low BRCA1 mRNA expression correlated with increased response rate (RR; *P* = 0.025 and 0.017, respectively) and median overall survival (mOS; *P* = 0.002 and *P*<0.001, respectively) in cisplatin-based chemotherapy or chemoradiotherapy group and also correlated with decreased RR (*P* = 0.017 and 0.024, respectively) and mOS (both *P*<0.001) in docetaxel-based chemotherapy or chemoradiotherapy group. Multivariate analysis revealed that low BRCA1 expression was an independent prognostic factor in cisplatin-based chemotherapy (HR 0.29; 95%CI 0.12–0.71; *P* = 0.007) or chemoradiotherapy (HR 0.12; 95%CI 0.04–0.37; *P*<0.001) group and higher risk for mortality in docetaxel-based chemotherapy (HR 5.02; 95%CI 2.05–12.28; *P*<0.001) or chemoradiotherapy (HR 7.02; 95%CI 2.37–27.77; *P*<0.001) group.

**Conclusions:**

BRCA1 mRNA expression could be used as a predictive and prognostic marker in esophageal cancer who underwent first-line cisplatin- or docetaxel-based treatments.

## Introduction

Esophageal cancer, as the sixth most common cause of cancer death in the world, lead to 407,000 deaths estimated in 2008 [1]. Patients with such an aggressive tumor have a poor 5-year survival rate less than 20%, which is most likely attributed to the presence of locally advanced and undetected metastatic disease at the time of diagnosis [2]. Chemotherapy has been playing important roles in improving survivals of esophageal cancer patients as part of multimodal therapy. Several chemotherapeutic agents commonly used in esophageal cancer have been investigated as single agent therapies with limited response rates as follows: 20% to 26% with cisplatin [3], 28% to 34% with paclitaxel [4] and 15% with 5-fluorouracil [5]. Combination chemotherapies with these chemotherapeutic agents appeared to be superior to single agent treatment with increased toxicity. Responsiveness to these chemotherapeutic agents and modalities varies among patients owing to genetic variations in pharmacokinetic and pharmacodynamic action [6]. The molecular backgrounds that determine therapeutic effectiveness in esophageal cancer still remain largely unknown. Some molecular markers have been identified for tailored treatment of esophageal cancer, including platinum related markers [glutathione S-transferase π (GST-π), excision repair cross-complementing 1 (ERCC1) and p-glycoprotein (P-gp)], 5-FU related markers [thymidylate synthase (TS) and dihydropyrimidine dehydrogenase (DPD)] and some proteins in various DNA repair pathways [7]–[9]. However, these studies mostly focused on the personalized therapies of 5-Fu or/and cisplatin-based treatments.

Breast cancer susceptibility gene 1 (BRCA1) was firstly identified as one of the genes that conferred genetic predisposition to early-onset breast and ovarian cancer [10]. However, due to its central component involved in multiple aspects of DNA damage responses and pivotal roles in the DNA repair pathway [10]–[13], increasing number of experimental and clinical investigations on BRCA1 as a regulator of chemotherapy-induced DNA damage have been performed [14]. The vast majority of cellular models have shown that BRCA1 upregulaion is notably associated with DNA repair mediated resistance to the DNA-damaging agent cisplatin through regulation of interstrand cross-link-induced premature senescence [15], nucleotide excision repair (NER) [16] and DNA double-strand-break homologous recombination (HR) repair pathway [17], [18]. Conversely, downregulation of BRCA1 confers resistance to antimicrotubule agent taxane through precocious inactivation of the spindle checkpoint [19], regulation of apoptotic pathways [20], [21] and transcriptional modifications of JNK signaling pathway [22]. It was also confirmed in clinic that BRCA1 mutations or alterations in BRCA1 mRNA and protein expression switch the response to cisplatin- or paclitaxel-based chemotherapy as well as influence the survivals in a number of malignancies, including breast cancer [23], [24], ovarian cancer [25]–[27], gastric cancer [28]–[30] and lung cancer [16], [31]. In our previous studies [29], [30], high levels of BRCA1 mRNA were negatively associated with cisplatin sensitivity but positively associated with docetaxel sensitivity in gastric cancer patients. Those advanced gastric carcinoma patients with high BRCA1 expression had significantly longer overall survivals compared to those with low expression (25.8 *vs* 9.5 months, *P* = 0.006) who received second-line docetaxel-based chemotherapy after first-line FOLFOX chemotherapy [29].

Cisplatin or docetaxel have been showed the most commonly and effectively utilized chemotherapeutic agents in clinic for patients with esophageal cancer [32]–[34]. However, no studies have addressed how variations in BRCA1 expressions influence clinical outcomes in patients with esophageal cancer treated with these chemotherapeutic agents. In this study, we firstly determined whether the levels of BRCA1 mRNA expression as predictive and prognostic biomarker were associated with clinical outcomes in esophageal cancer patients who received cisplatin- or docetaxel-based treatments.

## Patients and Methods

### Patients

A total of 155 patients with histologically confirmed locally advanced or metastatic esophageal squamous cell carcinoma (stage II-IV) and available paraffin-embedded tumor material for molecular analysis were enrolled in the study. They all had a better Eastern Cooperative Oncology Group performance status (PS; 0 to 2) and had measurable lesions. Among them 79 patients with surgically unresectable or metastatic disease received cisplatin- or docetaxel-based chemotherapy as the first-line treatment. The chemotherapy regimens comprised cisplatin-based regimens (cisplatin 25 mg/m2 on day 1–3 plus 5-fluorouracil 500 mg/m2 on day 1–5), docetaxel-based regimens (docetaxel 60–75 mg/m2 plus 5-fluorouracil 500 mg/m2 on day 1–5) and the combination with both (cisplatin 25 mg/m2 on day 1–3 plus docetaxel 60–75 mg/m2 on day 1). All chemotherapies were repeated every 3–4 weeks for a maximum of six cycles unless there was earlier evidence of disease progression or intolerable adverse effects. The other 76 patients with locally advanced disease received cisplatin or docetaxel-based concurrent chemoradiotherapy (CCRT) or radiotherapy alone as the first-line treatment. CCRT included chemotherapy and concurrent thoracic radiotherapy. The chemotherapy regimens consisted of weekly docetaxel (25 mg/m2 on day 1 per week) plus 5-fluorouracil (300 mg/m2 on day 1–3 per week) or cisplatin (25 mg/m2 on day 1 per week) plus 5-fluorouracil (300 mg/m2 on day 1–3 per week) for 5 weeks. The radiation dose was 50–60 grays (Gy) over 5 weeks (2 Gy/fraction per day, 5 fractions per week) with the use of CT simulation and 3 D treatment planning. Baseline and restaging assessment of measurable lesions were assessed by barium swallow and computed tomography scans, which was repeated every 2 cycle of chemotherapy or 4 weeks after radiotherapy.

The study was approved by the institutional ethics committee of Huai'an first people's hospital of Nanjing medical university and all patients gave their signed informed consent for the use of the tissue material in translational research.

### qPCR analysis for BRCA1 mRNA expression

We examined BRCA1 gene expression in paraffin-embedded specimens by biopsy under endoscope from the 155 patients as previously described [29]. Briefly, serial sections of 7-µm thickness with more than 80% tumor cells were prepared from primary tumor blocks by microdissection. Samples were lysed in a proteinase K-containing buffer after paraffin was removed by xylene. RNA was then extracted with phenol-chloroform-isoamyl alcohol followed by precipitation with isopropanol in the presence of glycogen and sodium acetate. RNA was resuspended in water and treated with DNAse I to avoid DNA contamination. cDNA was synthesized by using M-MLV retrotranscriptase enzyme. Template cDNA was amplified with specific primers and probes for BRCA1 and β-actin with using Taqman Universal Master Mix (Applied Biosystems, Foster City, CA). The primer and probe sets as reported in our previous study [29] were designed using Primer Express 2.0 Software (AB, Foster City, CA, USA). All primers and probes sequence were as follows: β-actin (NM_001101.3) forward 5′ TGAGCGCGGCTACAGCTT 3′, reverse 5′ TCCTTAATGTCACGCACGATTT 3′, and probe 6FAM -5′ACCACCA CGGCCGAGCGG 3′ TAMRA; BRCA1 (NM_007294) forward 5′GGCTATCCTCTCAGAGTGACATTTTA 3′, reverse 5′GCTTTATCAGGTTATGTTGCATGGT 3′, and probe 6FAM -5′CCACTCAGCAGAGGG 3′ MGB.

Quantitative real-time polymerase chain reaction (qPCR) was performed to quantify gene expression using the ABI Prism 7900HT Sequence Detection System (Applied Biosystems), which is more quantitative and accurate than immunohistochemistry used to assess protein expression as biomarker in clinical studies. The PCR conditions were 50°C for 2 minutes, 95°C for 10 minutes, followed by total 45 cycles at 95°C for 15 seconds and 60°C for 1 minute. Each sample was assayed in triplicate with commercial RNA as positive control and RNase-free water as negative control. In all quantitative experiments, only triplicates with a standard deviation (SD) of the quantification cycle (Cq) values of target genes less than 0.30 were accepted. Quantification of relative gene expression was performed according to the comparative Cq method using β-actin as an endogenous control. Gene expression analyses were conducted with the same calibrators throughout, specifically commercial human lung and liver RNA (Stratagene, La Jolla, CA, USA), to compare gene expression levels between different tumor types and between different cohorts of patients. Final values were determined by the formula 2^−ΔΔCq^ [ = 2^−(Cq sample - Cq calibrator)^] [29]. All analyses were performed at the molecular biology laboratory of Clinical Cancer Institute of Nanjing University (Nanjing, China).

### Study design and statistical analysis

The primary endpoint of the study was to examine the potential effects of BRCA1 mRNA expression levels on clinical responses and overall survival in esophageal cancer patients treated with cisplatin- or docetaxel-based chemotherapy or chemoradiotherapy in the first-line. Clinical responses were evaluated according to the Response Evaluation Criteria in Solid Tumors (RECIST) [35]. Overall survival was calculated from the date of diagnosis to the date of last follow-up or death from any cause. Progression-free survival was not examined because part of patients did not receive further assessments of disease after fist-line treatment and we could not get exact time of progress-free survival of these patients in the present retrospective study. Qualitative variables were summarized by absolute frequencies and percentages and quantitative variables were calculated in median values and ranges. BRCA1 expression values were divided into terciles and cutoff points were calculated according to the median value for the mRNA expression of BRCA1 [36]–[38]. The normality of quantitative variables was analyzed by the Kolmogorov-Smirnov test and compared with the Mann-Whitney U test. All statistical tests were two-sided. In order to correlate gene expression levels with clinical characteristics and compare categorical variables and response percentages, the two-sided chi-square test or Fisher's exact test was used for qualitative variables. The distributions of survival were obtained by the Kaplan-Meier method and compared with the two-sided log-rank test. A multivariate Cox proportional hazards regression model was performed to assess the association between each potential prognostic factor and survival.

All statistical analyses were performed with a power of 80% and at a 5% level of significance using the Statistical Package for the Social Sciences (SPSS) for Windows version 16 (SPSS Inc, Chicago, IL).

## Results

### Patients' characteristics

Clinical data and paraffin-embedded samples from the primary tumors were collected from 155 esophageal squamous cell carcinoma patients treated with cisplatin- or docetaxel-based chemotherapy/chemoradiotherapy in our centre. Successful amplification of BRCA1 gene was achieved in 144 specimens. The median age was 64; 90 patients were male and the majority of patients had PS 0-1. Among them, 72 patients treated with chemotherapy had stage III–IV and other 72 patients treated with chemoradiotherapy or radiotherapy alone had stage II–III at the time of diagnosis. In the chemotherapy group, the overall response rate (RR) was 38.9% and the median overall survival (mOS) was 11.3 months (95% CI, 9.0 to 13.6 months) after a median follow-up period of 10.6 months (range 3.0–30.0); while in the chemoradiotheapy group including those with radiotherapy alone, RR was 72.2% and mOS was 15.0 months (95% CI, 12.4 to 17.6 months) after a median follow-up period of 15.0 months (range 4.0–77.5). Patient characteristics are summarized in Table 1.

**Table 1:** Patient characteristics of advanced (stage II–IV) esophageal cancer patients.

		Chemotherapy72			Chemoradiotherapy72		
Characteristic	All patients	Cis/5-Fu	Doc/5-Fu	Cis/Doc	Radiotherapy alone	Radiotherapy+Cis/5-Fu	Radiotherapy+Doc/5-Fu
Patients, No. (%)	144 (100)	27 (18.8)	29 (20.1)	16 (11.1)	16 (11.1)	29 (20.1)	27 (18.8)
Age, y median (range)	63 (44–79)	60 (51–79)	62 (50–78)	61 (45–70)	69 (44–79)	63 (45–77)	64 (52–78)
Sex, No. (%)							
Male	90 (62.5)	17 (63.0)	16 (55.2)	12 (75.0)	8 (50.0)	19 (65.5)	18 (66.7)
Female	54 (37.5)	10 (37.0)	13 (44.8)	4 (25.0)	8 (50.0)	10 (34.5)	9 (33.3)
ECOG PS, No. (%)							
0–1	134 (93.1)	24 (88.9)	26 (89.7)	13 (81.2)	16 (100.0)	28 (96.6)	27 (100.0)
2	10 (6.9)	3 (11.1)	3 (10.3)	3 (18.8)	0 (0.0)	1 (3.4)	0 (0.0)
Stage, No. (%)							
II	31 (21.5)	0 (0.0)	0 (0.0)	0 (0.0)	9 (56.3)	14 (48.3)	8 (29.6)
III	45 (31.3)	2 (7.4)	1 (3.4)	1 (6.3)	7 (43.7)	15 (51.7)	19 (70.4)
IV	68 (47.2)	25 (92.6)	28 (96.6)	15 (93.7)	0 (0.0)	0 (0.0)	0 (0.0)
Site of tumor, No. (%)							
Upper	20 (13.9)	2 (7.4)	3 (10.3)	2 (12.5)	3 (18.8)	6 (20.7)	4 (14.8)
Middle	93 (64.6)	17 (63.0)	17 (58.6)	11 (68.8)	10 (62.5)	20 (70.0)	18 (66.7)
Lower	31 (21.5)	8 (29.6)	9 (31.0)	3 (18.8)	3 (18.8)	3 (10.3)	5 (18.5)
BRCA1 median (range)	11.96 (0.39–70.03)	10.63 (0.39–70.03)	8.69 (0.56–44.94)	13.58 (4.56–41.93)	10.23 (0.43–44.17)	11.39 (1.16–32.45)	14.37 (2.03–62.90)
Response rate (CR+PR), No. (%)		28 (38.9)			52 (72.2)		
Median OS (months, 95% CI)		11.3 (9.0–13.6)			15.0 (12.4–17.6)		

BRCA1, breast cancer susceptibility gene 1; Cis, cisplatin; 5-Fu, 5-fluorouracil; Doc, docetaxel; CR, complete response; PR, partial response; OS, overall survival; CI, confidence interval; ECOG, Eastern Cooperative Oncology Group; PS, performance status; y, years.

### BRCA1 mRNA expression levels

BRCA1 mRNA expressions were detected successfully in total 144 tumor samples by using quantitative real-time PCR and the median mRNA expression levels of BRCA1 relative to reference gene of β-actin were 11.96 (range 0.39–70.03). Patients were subdivided into two groups based on low (0.39–11.75; mean, 6.60) and high (12.17–70.03; mean, 23.90) levels of BRCA1 mRNA expression using a cutoff value of 11.96. The number of patients with low or high expression levels of BRCA1 was 72 in each cohort. There were no significant correlations between BRCA1 expression levels and clinical characteristics, including age (*P* = 0.317), gender (*P* = 0.388), performance status (*P* = 1.0), stage (*P* = 0.798), grade (*P* = 0.583) or site of tumor (*P* = 0.778) (Table 2).

**Table 2:** Clinical factors associated with BRCA1 mRNA expression levels.

	BRCA1 expression levels			Overall survival	
Characteristic	low	high	*P* value	MST (Months, 95% CI)	*P* Log-rank test
Age, No. (%)					
≤63	34	40		12.0 (10.0–14.0)	
>63	38	32	0.317	14.5 (12.8–16.2)	0.135
Sex, No. (%)					
Male	48	43		12.0 (10.0–14.0)	
Female	24	29	0.388	16.0 (13.2–18.8)	0.182
ECOG PS, No. (%)					
0–1	67	67		14.0 (12.5–15.5)	
2	5	5	1.000	6.1 (5.0–7.2)	<0.001
Stage, No. (%)					
II	15	17		16.0 (13.1–18.9)	
III	21	23		13.0 (11.1–14.9)	
IV	36	32	0.798	11.3 (9.6–13.0)	0.002
Grade, No. (%)					
G1	11	7		18.0 (11.1–24.9)	
G2	40	44		12.0 (9.8–14.2)	
G3	21	21	0.583	14.0 (11.0–17.0)	0.077
Site of tumor, No. (%)					
Upper	9	11		12.8 (6.8–18.8)	
Middle	46	47		13.5 (11.4–15.6)	
Lower	17	14	0.778	13.5 (10.4–16.6)	0.763

**Figure 1:**
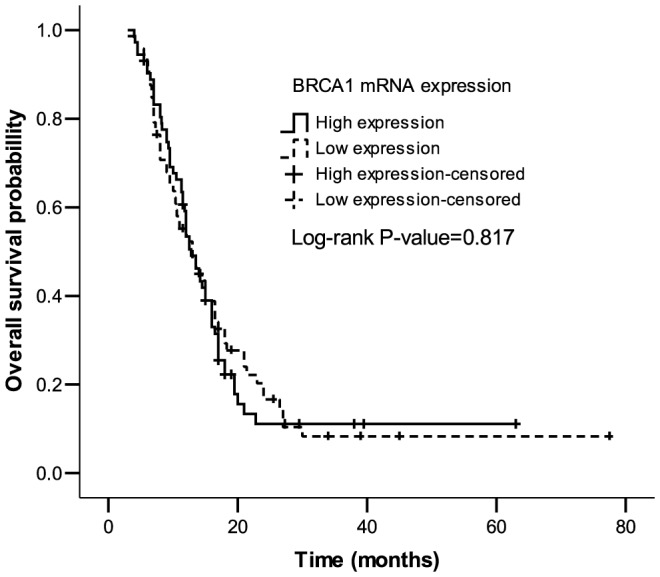
Median overall survival (mOS) in total 144 advanced and metastasis (stage II–IV) esophageal cancer patients receiving cisplatin- or docetaxel-based first-line treatments according to BRCA1 mRNA levels (for more details see **Table 1**).

BRCA1, breast cancer susceptibility gene 1; CI, confidence interval; MST, median survival time, ECOG, Eastern Cooperative Oncology Group; PS, Performance status;

In the whole cohort, 144 esophageal cancer patients had a mOS of 13.0 months (95% CI: 11.5–14.5 months), without statistical significance between the high and low expression of BRCA1 (13.0 *vs* 12.8, *P* = 0.817) (Figure 1).

### BRCA1 mRNA expression and treatment outcomes in chemotherapy group

**Figure 2:**
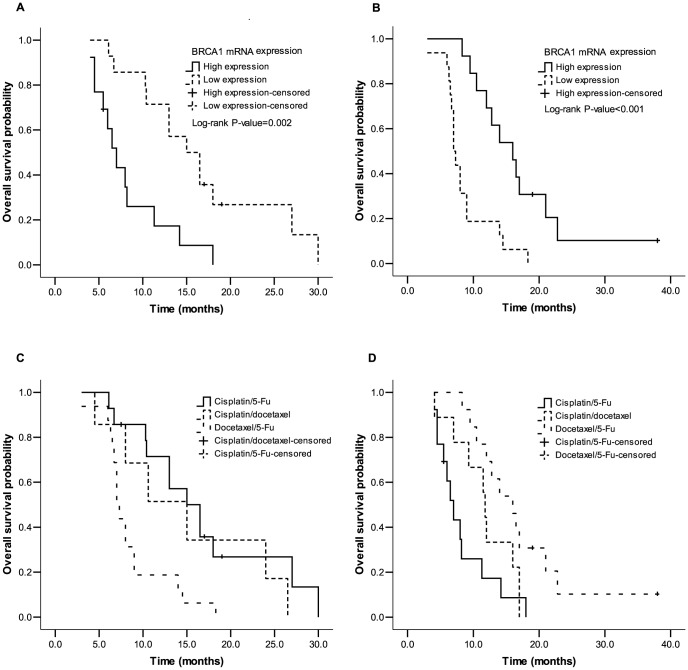
Median overall survival (mOS) in chemotherapy group: mOS in cisplatin/5-Fu (A) and docetaxel/5-Fu (B) subgroup according to BRCA1 mRNA levels (for more details see **Table 3**); mOS in low (C) and high (D) BRCA1 mRNA levels according to regimens of chemotherapy (for more details see **Table 4**).

In the chemotherapy group, patients with low BRCA1 expression had increased RR (57.1 *vs* 15.4%, *P* = 0.025) and mOS (15.0 *vs* 7.0, *P* = 0.002; Figure 2A) compared to those with high expression when treated with cisplatin-based chemotherapy; whereas when treated with docetaxel-based chemotherapy, patients with high BRCA1 mRNA expression had increased RR (69.2 *vs* 25.0%, *P* = 0.017) and mOS (16.0 *vs* 7.0, *P*<0.001; Figure 2B) compared to those with low expression (Table 3). Then we further investigated correlations of BRCA1 mRNA expression to clinical outcomes in chemotherapy group stratified by BRCA1 levels. Patients with low BRCA1 expression had the best clinical results when treated with cisplatin/5-Fu compared to docetaxel/5-Fu or cisplatin/docetaxel regimens [RR were 57.1, 25.0 (*P* = 0.073) and 28.6% (*P* = 0.217), respectively; mOS were 15.0, 7.0 (*P* = 0.002) and 15.0 months (*P* = 0.450), respectively; Figure 2C]. For those patients with high BRCA1 expression levels, regimen of docetaxel/5-Fu became the optimization choice in comparison with cisplatin/5-Fu or cisplatin/docetaxel regimens [RR were 69.2, 15.4 (*P* = 0.024) and 33.3% (*P* = 0.096), respectively; mOS were 16.0, 7.0 (*P* = 0.001) and11.8 months (*P* = 0.081), respectively; Figure 2D] (Table 4).

**Table 3:** Outcomes in different treatment groups according to BRCA1 expression levels.

		RR, *N* (%)			OS (months)		
Treatment	BRCA1	CR+PR (%)	SD+PD (%)	*P*-value	No. of patients	Median (95% CI)	*P*-value
Cis/5-Fu	Low	57.1	42.9	0.025	14	15.0 (10.7–19.3)	0.002
	High	15.4	84.6		13	7.0 (5.4–8.6)	
Doc/5-Fu	Low	25.0	75.0	0.017	16	7.0 (6.4–7.6)	<0.001
	High	69.2	30.8		13	16.0 (11.7–20.3)	
Cis/Doc	Low	28.6	71.4	0.635	7	15.0 (7.2–22.8)	0.220
	High	33.3	66.7		9	11.8 (10.1–13.5)	
Radiotherapy alone	Low	72.7	27.3	0.516	11	15.0 (9.2–20.8)	0.839
	High	60.0	40.0		5	12.5 (2.8–22.2)	
Radiotherapy+Cis/5-Fu	Low	87.5	12.5	0.017	16	24.0 (13.0–35.0)	<0.001
	High	46.2	53.8		13	11.3 (9.1–13.5)	
Radiotherapy+Doc/5-Fu	Low	50.0	50.0	0.024	8	7.5 (6.1–8.9)	<0.001
	High	89.5	10.5		19	19.5 (13.9–25.1)	

BRCA1, breast cancer susceptibility gene 1; Cis, cisplatin; 5-Fu, 5-fluorouracil; Doc, docetaxel; CR, complete response; PR, partial response; SD, stable disease; PD, progress disease; RR, response rate; OS, overall survival; CI, confidence interval; ECOG, Eastern Cooperative Oncology Group; PS, performance status;

**Table 4:** Outcomes in low or high BRCA1 expression levels according to regimens.

		RR, N (%)			OS (months)		
BRCA1	Treatment	CR+PR (%)	SD+PD (%)	*P*-value	No. of patients	Median (95% CI)	*P*-value
Low	Cis/5-Fu	57.1	42.9	1.000	14	15.0 (10.7–19.3)	1.000
	Doc/5-Fu	25.0	75.0	0.073	16	7.0 (6.4–7.6)	0.002
	Cis/Doc	28.6	71.4	0.217	7	15.0 (7.2–22.8)	0.450
High	Doc/5-Fu	69.2	30.8	1.000	13	16.0 (11.7–20.3)	1.000
	Cis/5-Fu	15.4	84.6	0.024	13	7.0 (5.4–8.6)	0.001
	Cis/Doc	33.3	66.7	0.096	9	11.8 (10.1–13.5)	0.081
Low	Radiotherapy+Cis/5-Fu	87.5	12.5	1.000	16	24.0 (13.0–35.0)	1.000
	Radiotherapy+Doc/5-Fu	50.0	50.0	0.046	8	7.5 (6.1–8.9)	<0.001
	Radiotherapy alone	72.7	27.3	0.332	11	15.0 (9.2–20.8)	0.070
High	Radiotherapy+Doc/5-Fu	89.5	10.5	1.000	19	19.5 (13.9–25.1)	1.000
	Radiotherapy+Cis/5-Fu	46.2	53.8	0.007	13	11.3 (9.1–13.5)	<0.001
	Radiotherapy alone	60.0	40.0	0.116	5	12.5 (2.8–22.2)	0.179

BRCA1, breast cancer susceptibility gene 1; Cis, cisplatin; 5-Fu, 5-fluorouracil; Doc, docetaxel; CR, complete response; PR, partial response; SD, stable disease; PD, progress disease; RR, response rate; OS, overall survival; CI, confidence interval; ECOG, Eastern Cooperative Oncology Group; PS, performance status;

### BRCA1 mRNA expression and treatment outcome in chemoradiotherapy group

**Figure 3:**
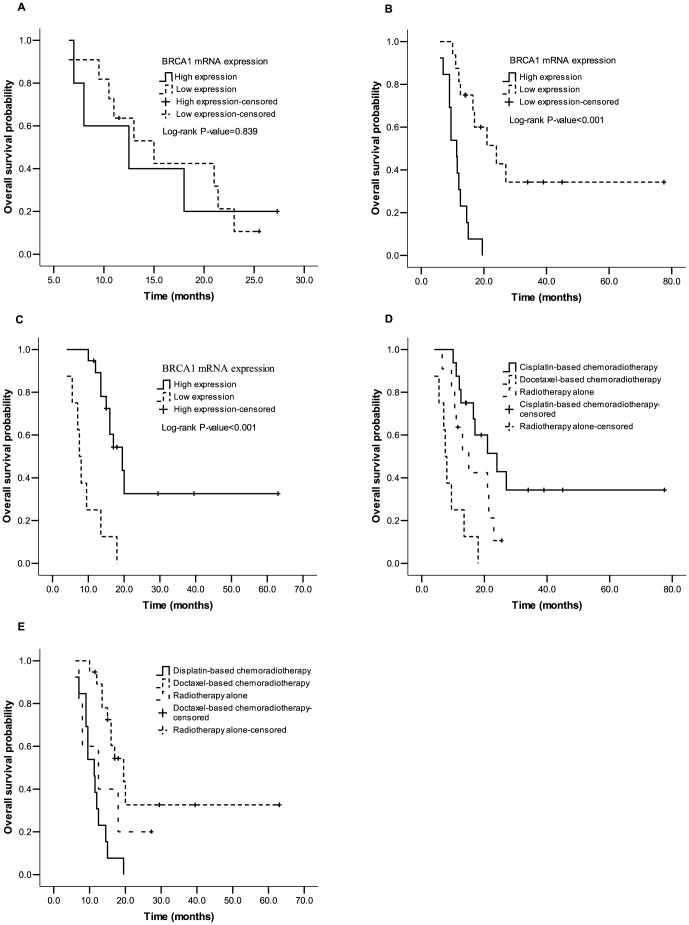
Median overall survival (mOS) in chemoradiotherapy group: mOS in radiotherapy alone (A), cisplatin-based chemotherapy (B) and docetaxel-based chemotherapy (C) subgroup according to BRCA1 mRNA levels (for more details see **Table 3**); mOS in low (D) and high (E) BRCA1 mRNA levels according to regimens of chemoradiotherapy (for more details see **Table 4**).

No significant differences were observed in RR (72.7 *vs* 60.0%, *P* = 0.516) and mOS (15.0 *vs* 12.0 months, *P* = 0.839; Figure 3A) between patients with low and high BRCA1 expression who were treated with radiotherapy alone. Nevertheless, when treated with concurrent chemoradiotherapy, patients with low BRCA1 expression had increased RR (87.5 *vs* 46.2%, *P* = 0.017) and mOS (24.0 *vs* 11.3 months, *P*<0.001; Figure 3B) compared to those with high expressions in the cisplatin-based chemoradiotherapy subgroup; and those with high BRCA1 expression had increased RR (89.5 *vs* 50.0%, *P* = 0.024) and mOS (19.5 *vs* 7.5 months, *P*<0.001; Figure 3C) compared to those with low expressions in the docetaxel-based chemoradiotherapy subgroup (Table 3). Further study on correlations of BRCA1 expression to clinical outcomes stratified by the BRCA1 expression suggested that cisplatin-based chemoradiotherapy was the best choice of treatment for patients with low BRCA1 expressions compared with treatment of docetaxel-based chemoradiotherapy or radiotherapy alone [RR were 87.5, 50.0 (*P* = 0.046) and 72.7% (*P* = 0.332), respectively; mOS were 24.0, 7.5 (*P*<0.001) and 15.0 months (*P* = 0.070), respectively; Figure 3D]; whereas, docetaxel-based chemoradiotherapy was the best one for those with high BRCA1 expressions compared with cisplatin-based chemoradiotherapy or radiotherapy alone [RR were 89.5, 46.2 (*P* = 0.007) and 60.0% (*P* = 0.116), respectively; mOS were 19.5, 11.3 (*P*<0.001) and 12.5 months (*P* = 0.179), respectively; Figure 3E] (Table 4).

### Univariate and multivariate analyses

Univariate analysis demonstrated that a significant association was observed between mOS and PS (*P*<0.001) or stage of disease (*P* = 0.002) in the whole cohort. No other association between clinical characteristics and survival was found (Table 2).

Cox proportional hazard analysis revealed that low BRCA1 expression as an independent prognostic factor was significant associated with increased mOS in cisplatin-based chemotherapy (HR 0.29; 95%CI 0.12–0.71; *P* = 0.007) or chemoradiotherapy (HR 0.12; 95%CI 0.04–0.37; *P*<0.001) group, whereas low BRCA1 expression emerged conversely as higher risk for mortality associated with decreased mOS in docetaxel-based chemotherapy ( HR 5.02; 95%CI 2.05–12.28; *P*<0.001) or chemoradiotherapy (HR 7.02; 95%CI 2.37–27.77; *P*<0.001) group (Table 5).

**Table 5:** Multivariate analysis of factors associated with overall survival of esophageal cancer according to regimens.

	Cis/5-Fu			Doc/5-Fu			Radiotherapy+Cis/5-Fu			Radiotherapy+Doc/5-Fu		
	HR.	CI,	*P*	HR.	CI,	*P*	HR.	CI	*P*	HR.	CI,	*P*
BRCA1 (low *vs* high)	0.29	0.12–0.71	0.007	5.02	2.05–12.28	<0.001	0.12	0.04–0.37	<0.001	7.02	2.37–27.77	<0.001
Stage (II vs III or III vs IV)	0.23	0.03–1.76	0.156	4.39	0.51–38.03	0.179	1.72	0.71–4.18	0.232	1.14	0.37–3.45	0.823
PS (0–1 *vs* 2)	0.33	0.08–1.37	0.127	0.13	0.03–0.54	0.005	0.47	0.004–0.51	0.012	-	-	-

BRCA1, breast cancer susceptibility gene 1; HR, hazard ratio; CI, confidence interval; *P, P*-value.

## Discussion

In present study, we firstly applied qPCR analysis of BRCA1 mRNA expression in formalin-fixed paraffin-embedded tumor tissue in advanced and metastasis esophageal cancer and evaluated the relationship between BRCA1 mRNA expression levels and cisplatin- or docetaxel-based treatments. The antimetabolite 5-fluorouracil was used as combination agent in cisplatin- or docetaxel-based treatment because BRCA1 failed to modulate resistance or sensitivity to it [21]. BRCA1 was discovered to be involved in the inverse resistance relationship between cisplatin- and docetaxel-based treatments in esophageal cancer patients. Over expression of BRCA1 mRNA was negatively associated with RR and mOS in patients treated with cisplatin-based chemotherapy or chemoradiotherapy; whereas conversely, over expression of BRCA1 was positively associated with clinical outcomes in those patients who received docetaxel-based treatments. Multivariate analysis revealed that low BRCA1 expression was an independent prognostic factor for patients with cisplatin-based treatment and conversely higher risk for mortality for patients with docetaxel-based treatment. These findings support the hypothesis that BRCA1 mRNA expression levels could be a valid indicator of differential sensitivity to cisplatin or docetaxel in esophageal cancer, which is consistent with findings in previous clinical studies on other malignancies [23]–[31]. As a predictive marker in previous studies, overexpression of BRCA1 was significantly correlated with higher RR and progression-free survival (PFS) but not with mOS in non-small-cell lung cancer (NSCLC) treated with docetaxel-gemcitabine as first-line chemotherapy [36]; BRCA1 mutation appeared to be related with high pathologic complete response in breast cancer treated with platinum-based neoadjuvant therapy [23], [24]. Meanwhile, as a prognostic marker, low BRCA1 expression correlated with improved survival in advanced ovarian cancer who received platinum-based chemotherapy [27] and high BRCA1 expression correlated with longer mOS in gastric cancer patients treated with second-line docetaxel-based chemotherapy after first-line FOLFOX chemotherapy [29]. As both predictive and prognostic marker, low BRCA1 protein expression correlated with better clinical outcome in terms of both PFS and OS in patients with epithelial ovarian cancer treated with platinum [25], [26]. In the present study, BRCA1 mRNA determined by qPCR with merits of better quantitative and accurate measurement compared to protein expression determined by immunohistochemistry, was found to be both predictive marker associated with RR and prognostic marker associated with mOS in esophageal cancer patients treated with docetaxel- or cisplatin-based treatments.

These results are also in line with findings in the pre-clinical cell line model, which indicated that reconstitution of wild-type BRCA1 function into human breast cancer HCC1937 cell line caused a 1000-fold increase in sensitivity to taxane and a 20-fold increase in resistance to cisplatin [21]. Several mechanisms were involved in the resistance of DNA-damaging agent cisplatin [15]–[18] as mentioned previously. Among them, NER is considered as the main mechanism for the resistance of cisplatin through removal of the cisplatin-DNA adducts that mainly cause cellular death and tumor response [38]. ERCC1 involved in GG-NER pathway has been proved an effective predictive marker of cisplatin response [39]. By contrast, BRCA1 may be a better predictive marker of cisplatin response owing to its involvement in TC-NER that is relevant for the antiproliferative activity of cisplatin [16]. Meanwhile, downregulation of BRCA1 confers resistance to taxane through inactivation of the spindle checkpoint, regulation of apoptotic and JNK signaling pathways [19]–[22].

Cisplatin- and docetaxel-based treatments were found to be the optimized choices for patients with low and high expression of BRCA1 to get more clinical benefits in the present study, respectively. Survival benefits can be brought to esophageal caner patients by appropriate treatments based on BRCA1 expression. In this retrospective study, patients with stage III–IV had longer mOS of 15.0 months (95% CI: 10.7–19.3) when treated with cisplatin-based chemotherapy for low BRCA1 expression and 16.0 months (95% CI: 11.7–20.3) when treated with docetaxel-based chemotherapy for high BRCA1 expression. By contrast, previous phase II clinical trial indicated that esophageal cancer patients with similar stages only had mOS of 10.8 months when treated with a combination therapy of three chemotherapeutic agents including paclitaxel, cisplatin and 5-FU [33]. Meanwhile, no clinical benefits were observed in patients with low BRCA1 expression who received docetaxel-based treatments and patients with high BRCA1 expression who received cisplatin-based treatments (Table 4). Patients with low BRCA1 expression treated with cisplatin/docetaxel chemotherapy had similar OS (15.0 *vs* 15.0 months, *P* = 0.450, Figure 2C) compared to cispalin/5-Fu chemotherapy and patients with high BRCA1 expression treated with cisplatin/docetaxel chemotherapy also had no better OS (11.8 vs 16.0 months, P = 0.081, Figure 2D) compared to docetaxel/5-Fu chemotherapy (Table 4). These results were consistent with findings in previous study on NSCLC that overexpression of BRCA1 was significantly correlated with better clinical outcomes in patients treated with docetaxel/gemcitabine but not in those treated with cisplatin/docetaxel regimen [36].

Radiotherapy is considered extremely effective in local control of cancer, but its curative potential is often limited by intrinsic radioresistance of the tumor cells. BRCA1 is involved in HR repair of double strands break with relation to radiation resistance and considered important in maintenance of genomic stability through DNA repair while cell DNA-damage takes place due to radiation [17], which is supported by the observation that cells deficient in BRCA1 are highly sensitive to radiation [40]. Nevertheless, we didn't observe the effect of BRCA1 on resistance of radiotherapy for patients with esophageal cancer. In present study, there was no significant difference in clinical outcomes between patients with low and high BRCA1 expression when treated with radiotherapy alone. This result was in agreement to the previous study of breast cancer, which showed no evidence of increased radiation sensitivity in breast tissue heterozygous for a BRCA1/2 germline mutation [23]. This finding might also explain why BRCA1 mRNA expression was significantly associated to cisplatin- or docetaxel-based concurrent chemoradiotherapy in the same trend that existed in chemotherapy group.

Several limitations exist in this study which is based on an unplanned and retrospective analysis, lack of validation group and has relatively small number of patients in treatment subgroups. However, we provide the first evidence to support the tumor mRNA expression levels of BRCA1 as predictive and prognostic marker in esophageal cancer with cisplatin- or docetaxel-based treatments and indicated the need to further validate in a large number of patients prospectively.
